# Lexicality-Modulated Influence of Auditory Cortex on Subthalamic Nucleus During Motor Planning for Speech

**DOI:** 10.1162/nol_a_00086

**Published:** 2023-01-18

**Authors:** Alexander R. Weiss, Anna Korzeniewska, Anna Chrabaszcz, Alan Bush, Julie A. Fiez, Nathan E. Crone, Robert M. Richardson

**Affiliations:** JHU Cognitive Neurophysiology and BMI Lab, Department of Neurology, Johns Hopkins University School of Medicine, Baltimore, MD, USA; Department of Psychology, University of Pittsburgh, Pittsburgh, PA, USA; Brain Modulation Lab, Department of Neurosurgery, Massachusetts General Hospital, Boston, MA, USA; Harvard Medical School, Boston, MA, USA; Department of Communication Science and Disorders, University of Pittsburgh, Pittsburgh, PA, USA; University of Pittsburgh Brain Institute, Pittsburgh, PA, USA

**Keywords:** event related causality, subthalamic nucleus, basal ganglia, large-scale network interactions, human EEG, electrophysiology

## Abstract

Speech requires successful information transfer within cortical-basal ganglia loop circuits to produce the desired acoustic output. For this reason, up to 90% of Parkinson’s disease patients experience impairments of speech articulation. Deep brain stimulation (DBS) is highly effective in controlling the symptoms of Parkinson’s disease, sometimes alongside speech improvement, but subthalamic nucleus (STN) DBS can also lead to decreases in semantic and phonological fluency. This paradox demands better understanding of the interactions between the cortical speech network and the STN, which can be investigated with intracranial EEG recordings collected during DBS implantation surgery. We analyzed the propagation of high-gamma activity between STN, superior temporal gyrus (STG), and ventral sensorimotor cortices during reading aloud via event-related causality, a method that estimates strengths and directionalities of neural activity propagation. We employed a newly developed bivariate smoothing model based on a two-dimensional moving average, which is optimal for reducing random noise while retaining a sharp step response, to ensure precise embedding of statistical significance in the time–frequency space. Sustained and reciprocal neural interactions between STN and ventral sensorimotor cortex were observed. Moreover, high-gamma activity propagated from the STG to the STN prior to speech onset. The strength of this influence was affected by the lexical status of the utterance, with increased activity propagation during word versus pseudoword reading. These unique data suggest a potential role for the STN in the feedforward control of speech.

## INTRODUCTION

Speech requires the precise coordination of vocal articulators by specialized brain areas (Bouchard et al., 2013; Collard et al., 2016; Conant et al., 2014). Evidence that articulation is disrupted in neurological disorders affecting the basal ganglia strongly implicates these regions in the motor aspects of speech production. For example, impairments like hypophonia and hypokinetic dysarthria are present in up to 90% of patients with Parkinson’s disease (PD; Illes et al., 1988; Liotti et al., 2002; Metter & Hanson, 1986; Miller et al., 2007). Other studies indicate roles for the basal ganglia in linguistic processes beyond motor control of speech such as lexical retrieval, verbal fluency, speech pitch and speed variation, action-verb naming, and the comprehension of syntax and grammar (Duffy, 2012; Grossman et al., 1992, 1993, 2012; Hochstadt, 2009; Péran et al., 2009; Simard et al., 2011; Terzi et al., 2005; Yarnall et al., 2014; Yu et al., 2010). In fact, deep brain stimulation (DBS) of the subthalamic nucleus (STN) has been shown to decrease both semantic and phonological fluency in patients with PD, directly implicating the basal ganglia in linguistic processing (Deep-Brain Stimulation for Parkinson’s Disease Study Group, 2001; Kleiner-Fisman et al., 2006; Klostermann et al., 2008; Skodda, 2012; Vos et al., 2021). Other studies have reported improvements in speech function following DBS that depend on lead location within the STN (Jorge et al., 2020). Better understanding the role of the basal ganglia in speech production will improve neurolinguistic models and models of speech motor control and may lead to more effective speech treatments for related neurological diseases (Smith & Caplan, 2018).

The parallel circuit model posits that information from diverse areas of the cortex responsible for sensorimotor, associative, and limbic processes progress through anatomically and functionally distinct cortical-basal ganglia loops (Alexander et al., 1986; Moore & Bloom, 1978). Broad areas of the cortex send excitatory projections to the striatum, the primary input nuclei of the basal ganglia, where neural communication diverges into direct and indirect pathways to reach the primary output nuclei of the basal ganglia (Harris et al., 2019). A third pathway, the hyperdirect pathway, carries cortical inputs directly to the STN and has been implicated in behaviors such as action selection, action focusing, and motor learning (Desmurget & Turner, 2010; Nambu et al., 2002; Turner & Desmurget, 2010; Wichmann et al., 1994; Zaghloul et al., 2012). New evidence from mapping cortical evoked potentials generated through stimulating the STN suggests the presence of a sensory hyperdirect pathway from the superior temporal gyrus (STG) to STN, positioning the STN to contribute to the integration of sensory and motor information in the control of speech (Jorge et al., 2022).

Intraoperative recordings collected during DBS implantation surgery offer a unique opportunity to directly assess functional connections between the STG and STN during speech production. To this end, we analyzed the dynamics of activations during an overt reading task, in simultaneous recordings from STN, the ventral sensorimotor cortex, and posterior STG. Words and pseudowords were analyzed to investigate whether the influence of the STN varied as a result of lexicality (Deep-Brain Stimulation for Parkinson’s Disease Study Group, 2001; Kleiner-Fisman et al., 2006; Klostermann et al., 2008; Skodda, 2012; Woolnough et al., 2022). We analyzed the dynamics of these activations during an overt reading task, in simultaneous recordings from STN, the ventral sensorimotor cortex, and posterior STG. Words and pseudowords were used to investigate whether the influence of the STN varied as a result of lexicality (Deep-Brain Stimulation for Parkinson’s Disease Study Group, 2001; Kleiner-Fisman et al., 2006; Klostermann et al., 2008; Skodda, 2012; Woolnough et al., 2022).

We used the event-related causality (ERC) technique to provide an estimate of the directionality, intensity, and frequency content of task-related interactions between neural recording sites (Korzeniewska et al., 2008). The ERC technique hinges upon the idea that distributed neuronal sites can become causally interacting through the propagation of their oscillatory activity. This is because oscillations provide an effective means of controlling the timing of neuronal firing, allowing the temporal coordination of information transfer across brain regions (Buzsáki & Draguhn, 2004; Engel et al., 2001; Fries, 2005; Varela et al., 2001). Even single neurons are endowed with these complex dynamics, and their intrinsic ability to resonate and oscillate at multiple frequencies suggests that the precise timing of their oscillatory activity within neuronal networks represents information (Buzsáki & Draguhn, 2004; Engel & Fries, 2010; Hutcheon & Yarom, 2000; Llinás, 1988). This information can be processed and transferred by flexible cell assemblies, defined as distributed networks of neuronal groups, that are transiently synchronized by dynamic connections (Engel et al., 2001; Varela et al., 2001). The ability of neuronal assemblies to synchronize depends on the coupling strength and the distribution of natural frequencies and are the result of the physical architecture of neuronal networks. These network oscillations bias input selection and temporally link neurons into assemblies (Buzsáki & Draguhn, 2004; Engel & Fries, 2010; Nunez, 1995). The synchronous activity of oscillating networks is viewed as the critical “middle ground” linking single-neuron activity to behavior (Engel et al., 2001; Hasselmo et al., 2002; Somers & Kopell, 1993; Steriade, 2001; Traub et al., 1999; Whittington & Traub, 2003). Indeed, cognitive function results from synchronized networks (Engel et al., 2001; Gray et al., 1989; Kahana et al., 2001; Llinás & Ribary, 1993; Varela et al., 2001).

In order to study integration through synchronization, one needs to focus on the temporal dynamics of neural networks in the millisecond range synchronization (Varela et al., 2001). For this reason, we applied the ECR to the 60–180 Hz frequency (high-gamma) range of neuronal oscillations. As high-gamma oscillations represent a general index of neuronal population firing rates, they are perfectly suited for studying the dynamics of subcortical-cortical network interactions (Ray et al., 2008). Analysis of the fine network dynamics measured by ERC during word production tasks previously showed that perisylvian language sites (including the middle temporal gyrus and STG) interact with different areas of the sensorimotor cortex dependent on the modality of speech, suggesting that the excellent time–frequency resolution of ERC should well capture the dynamics of cortical-STN communication during word production (Alhourani et al., 2020; Flinker et al., 2015; Korzeniewska et al., 2011). Consistent with this expectation, neuronal oscillations, as increases in neuronal oscillations within this frequency range have been observed in the cortex during motor and word production tasks and in STN during movement initiation and inhibition. Neural activity in this frequency range also displays coherence between STN and motor cortex. These considerations strongly suggest that subcortical-cortical communications in this frequency range influence speech production (Benítez-Burraco & Murphy, 2019; Brücke et al., 2008; Crone, Boatman, et al., 2001; Crone et al., 1998; Crone, Hao, et al., 2001; Fischer et al., 2020; Jenkinson et al., 2013; Lachaux et al., 2012; Muthukumaraswamy, 2010; Pantev, 1995; Ray et al., 2003, 2008). The current study complements this past work through its novel examination of relationships between areas implicated in sensory aspects of speech and the STN during the overt reading of words and pronounceable nonwords. Our recent modification of the ERC method ensured precise embedding of the results in the time–frequency space (Korzeniewska et al., 2022), allowing us to distinguish between speech planning and production epochs of the task. Our findings contribute important new information related to the integration of sensory and motor information in the control of speech production.

## MATERIALS AND METHODS

### Subjects

Subjects were native English speakers with PD undergoing awake stereotactic neurosurgery for implantation of DBS electrodes in the STN, as recommended by a clinical multidisciplinary movement disorders surgery board. All subjects provided informed consent prior to surgery in order to participate in the study. This study was conducted according to a protocol approved by the University of Pittsburgh Medical Center Internal Review Board (IRB Protocol # PRO13110420).

In addition to clinical subcortical mapping, subjects were implanted with subdural electrode arrays over the left lateral sensorimotor cortex that were removed after intraoperative task completion. The safety of this research technique has been demonstrated in over 500 patients (Panov et al., 2017; Sisterson et al., 2021). It was the surgeon’s standard procedure to implant the left DBS lead first. Language dominance laterality was not determined pre-operatively, although it is typically assumed that most people (including non-right-handed subjects) have left-hemisphere language lateralization (Knecht et al., 2000; Szaflarski et al., 2002).

Recordings from 11 patients were subjected to screening for appropriateness for the ERC analysis, including suitable cortical and STN electrode coverage and significant task-related high-gamma frequency local field potential (LFP) modulation. Data from 10 recording sessions across four subjects (3 male/1 female, age: 70.25 ± 3.94 years old, duration of disease: 7.50 ± 0.43 years, mean ± *SEM*) who met the criteria for ERC analysis were investigated. All subjects completed Unified Parkinson’s Disease Rating Scale (UPDRS; Goetz et al., 2007) testing within a four-month period prior to DBS implantation surgery. Dopaminergic medication was withdrawn the night before surgery. Subjects’ demographic and clinical characteristics are provided in Table 1.

**Table 1.** Demographic and clinical characteristics of patients included in the study.

Subject	Gender	Age	Handedness	Education (yr)	Duration of disease (yr)	Hoehn and Yahrstage^a^	UPDRS speech score^b^ (off medication)	UPDRS score total (off medication)
1	Male	68	Left	16	8	2	1	50
2	Male	82	Right	16	8	2	2	36
3	Female	71	Right	16	8	2	1	24
4	Male	60	Right	13	6	2	1	39
Mean ± *SE*	–	70.25 ± 3.94	–	15.25 ± 0.65	7.50 ± 0.43	2.00 ± 0.00	1.25 ± 0.22	37.25 ± 4.63

*Note*. UPDRS = Unified Parkinson’s Disease Rating Scale.

^a^ Goetz et al. (2004).

^b^ Goetz et al. (2007).

### Behavioral Paradigm

Subjects performed the overt reading task during surgical pauses in the subcortical mapping of the STN on the first (left) side, in up to four recording sessions (each reflecting a different depth within the STN) per patient. Each session was composed of 60 trials with alternating words and pseudowords.

Visual stimuli were created and presented by custom code running in the MATLAB environment (MathWorks, 2018) using Psychophysics Toolbox extensions (Brainard, 1997). Stimuli consisted of single consonant-vowel-consonant (CVC) words or pseudowords presented on a computer screen and subjects were familiarized with the task before undergoing surgery (Chrabaszcz et al., 2019). A total of 240 stimuli (120 words and 120 phonotactically legal pseudowords) were constructed. To control for articulatory complexity and prevent potential learning effect confounds from repeated testing sessions, four presentation lists were constructed across which stimuli were pseudorandomized and balanced along a number of psycholinguistic parameters, such as phonological and orthographic neighborhood density, bigram frequency, and phonotactic and biphone probability (Moore et al., 2017). On each trial, subjects were presented with a white cross against a black background during an intertrial interval, after which a green fixation cross (preparatory cue) appeared on the screen for 250 ms instructing the subject to get ready. This was followed by a variable interstimulus interval (ISI; 500–1,000 ms) during which the screen remained black. Following the ISI delay, the stimulus was presented on the screen and subjects were instructed to read it out loud. The stimulus remained on the screen until subjects made their response, after which the experimenter manually advanced the presentation to the next trial. Subjects were instructed to respond as quickly as they could following each stimulus appearance. Overt pronunciation of words and pseudowords was chosen to provide online speech onset timing and error data (allowing us to remove error trials) and to confirm that targeted psycholinguistic effects were indeed present (Indefrey, 2011). Speech onset for word trials, speech onset for pseudoword trials, and speech onset for both word and pseudoword trials together (referred to as “pooled trials”) defined epochs of interest that were used in ERC analysis.

### Subthalamic Nucleus and Cortical Recordings

Subjects were awake and anesthetic agents had been withheld for a period of at least 30 min prior to the performance of the overt reading task. No additional medications were delivered while subjects performed the task.

Subjects were temporarily implanted with subdural electrode arrays over the cortical surface of the left hemisphere, which were passed through the existing surgical burr hole after the dura was opened, but prior to the insertion of micro-electrode guide tubes. Burr hole locations were determined solely by the standard clinical procedure of selecting the safest entry point for the intended DBS trajectory. Subject 1 and Subject 4 were implanted with 6- and 28-channel PMT (2022) electrode strips, respectively. Subject 2 and Subject 3 were implanted with 54- and 36-channel Ad-Tech Medical (2022) electrode strips, respectively. Electrode strip contact sizes were 1, 2, or 4 mm diameter and center-to-center spacing was 3, 4, or 10 mm. The placement of the electrode strips was targeted over ventral sensorimotor cortex by using stereotactic coordinates to mark the scalp over this region and advancing the subdural strips in the direction of this overlying visual marker.

A referential montage was used with the reference electrode placed in the scalp and a ground electrode placed on the skin overlying the acromion process. STN LFPs were recorded at a sampling rate of 44 kHz and bandpass filtered from 0.075 Hz to 10 kHz. The electrocorticographic (ECoG) signals was acquired at 30 kHz using the Grapevine–Neural Interface Processor (MathWorks, 2022).

The superior and inferior boundaries of the STN were determined by the neurophysiologist and neurosurgeon based on characteristic STN single-unit neuronal activity obtained from three microelectrodes simultaneously advanced through the STN in 0.1 mm increments. The dorsolateral STN was targeted for treatment of motor symptoms in PD, as previously described (Crammond & Richardson, 2020). LFP data were acquired for up to four different depths within the STN per patient during separate runs of the overt reading task, with each task session capturing three unique STN recording sites. According to the assumptions of the ERC method (described in detail below) we estimated a different ERC multivariate autoregression (MVAR) model to each session, and considered each session as having a distinct electrode set. As a result, LFP data from a total of 30 STN recording sites were obtained across all analyzed subjects, noting that for the most superficial recording sites within the STN, the electrode may have been just superior to the dorsal border of STN.

### Electrode Localization

The electrodes were localized using a custom method to align pre-operative MRI, intraoperative fluoroscopy (512 × 512 pixels, OEC 9900; General Electric, 2022), and post-operative computed tomography (CT), as described previously (Lipski et al., 2017; Randazzo et al., 2016). Briefly, CT and MRI scans were co-registered using mutual information in the Statistical Parametric Mapping (SPM12) software package (Ashburner et al., 2021) and rendered into 3D skull and brain surfaces using Osirix Version 7.5 and Freesurfer Version 5.3 software (Dale et al., 1999; Rosset et al., 2004). The co-registered images and fluoroscopy images were aligned using common landmarks: The subject’s stereotactic frame pins, implanted depth electrodes, and skull outline. Parallax effects in the fluoroscopic images were accounted for using the measured distance from the radiation source to the subject’s skull. Following surface-to-fluoroscopic image alignment, a 3D location for each electrode was projected from the fluoroscopic image onto the cortical surface, and based upon cortical parcellations for each subject’s anatomy, electrodes were assigned to a cortical gyrus (Alhourani et al., 2020; Desikan et al., 2006). Electrodes could then be grouped into anatomical regions, and only those localized to the precentral gyrus, postcentral gyrus, and STG were included for further analysis, in addition to STN contacts. Electrode locations were registered to a common brain space using the Brainstorm MNI ICBM152 template, which is documented and freely available for download online under the GNU general public license (https://neuroimage.usc.edu/brainstorm; Fonov et al., 2009, 2011; Nasiotis et al., 2019; Tadel et al., 2011).

**Figure 1.**
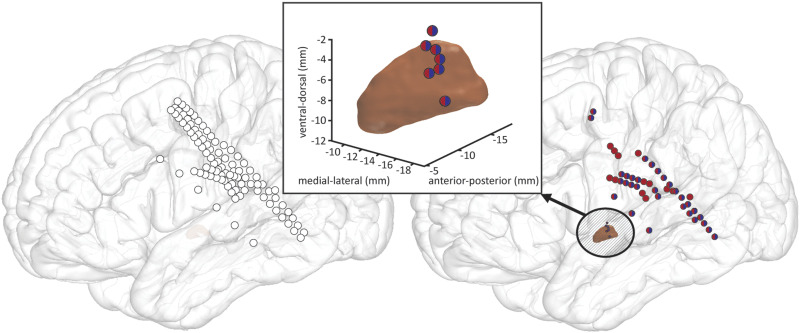
Recording sites in four studied patients during the behavioral paradigm. (Left panel) All recording sites, projected onto an MNI brain atlas. (Right panel) Sites demonstrating significant event-related high-gamma power augmentation in pseudoword (red), word (blue), and pooled pseudoword and word trials (vertically split red/blue). (Center insert) Sites demonstrating significant event-related high-gamma power augmentation in subthalamic nucleus (STN, the same color-coding).

DBS electrodes were semi-automatically localized and transformed into a standard template for group visualization using the Lead-DBS toolbox (Horn et al., 2019; Horn & Kühn, 2015). In brief, post-operative CT scans were linearly co-registered with pre-operative MRI scans and normalized to Montreal Neurological Institute (MNI) space. MNI-defined coordinates of electrode contact locations were extracted for all analyzed subjects, color-coded per stimuli-type, and visualized in Figure 1.

### Audio Recordings

Subjects’ vocal responses were recorded using an omnidirectional microphone (either Audio-Technica model ATR3350iS Mic, frequency response 50–18000 Hz; or PreSonus model PRM1 Precision Flat Frequency Mic, frequency response 20–20000 Hz). The microphone was positioned approximately 8 cm from the subject’s mouth and oriented at an angle of approximately 45 degrees. In the cases where the PreSonus PRM1 microphone was used, a Zoom H6 digital recorder was required to record the audio signal at a sampling rate of 96 kHz. In all cases, the signal was simultaneously recorded using a Grapevine–Neural Interface Processor (MathWorks, 2022) at a lower sampling rate of 30 kHz. The audio recordings were synchronized with visual cue events and with the neural recordings using digital pulses delivered to a Neuro-Omega system (Alpha Omega Engineering, n.d.) via a USB data acquisition unit (model USB-1208FS; Measurement Computing, 2022). Audio recordings were segmented and transcribed offline using the International Phonetic Alphabet in a custom designed graphical user interface implemented in MATLAB.

### Electrophysiological Data Preprocessing

Data processing was performed using the SPM12 (Ashburner et al., 2021) and Fieldtrip toolboxes implemented using custom scripts (Oostenveld et al., 2011). A subject’s recorded signal was aligned with the presentation of the green cross cue for baseline epoching, and with subject’s articulation of the initial consonant for speech response-aligned epoching. All trial epochs were visually inspected for any artifacts. Channels with extensive artifacts from movement, powerline, or environmental sources were visually identified and removed from further analyses, and any contaminated segments were rejected. Signals were re-referenced using a common average reference, which minimizes the contribution of components present in all signals, highlighting the interactions within the studied system. Signals were forward–backward band-pass filtered using a finite impulse response filter at 60–180 Hz to extract the high-gamma signal and to reassure zero-phase distortion. Filter characteristics were designed to capture high-gamma responses while avoiding line noises from 60 Hz and its harmonic.

### Data Selection

Trials were included in the analysis if it was possible to unambiguously identify a subject’s spoken response and a subject’s response included the stimuli’s targeted phonemes. On the basis of these criteria, seven (1.17%) out of a total of 600 recorded trials were rejected. Selection of LFP channels for ERC analysis was based on the results of spectral analyses demonstrating statistically significant event-related power augmentation in high-gamma frequencies (60–180 Hz) during the same task in the same patient, as determined using a two-sided *t* test comparing each time point post-stimulus presentation to the mean of all time points of baseline in each frequency (Crone, Hao, et al., 2001). Only electrodes that displayed significance in the above high frequency band using this method were eligible for ERC analysis. Using this method, out of a total of 124 electrodes placed on the cortex and 30 DBS recording sites across the analyzed subjects and sessions, 53 cortical sites significantly activated (42.7% of implanted electrodes) and 10 STN sites (33.3%) were significantly activated during speech response, for the pooled (word and pseudoword) trials. In separate word and pseudoword analyses, a total of 36 (29.0%) and 46 (37.1%) cortical electrodes, respectively, exhibited significant event-related high-gamma activity. Both word and pseudoword analyses each had 10 (33%) STN DBS sites with significant activity.

### Behavioral Data Analysis

Reading latency (i.e., the time required for subjects to start articulation from the time of stimulus onset to the time of speech onset) was calculated for all analyzed word and pseudoword trials. The time required for subjects to articulate the first consonant (C1), vowel (V), and second consonant (C2) of a given trial’s CVC stimuli was calculated from the recorded session audio, averaged across trials, sessions, and subjects. Statistical differences between reading latencies and CVC metrics were assessed using Wilcoxon signed-ranked tests.

### Event-Related Causality Analysis

To evaluate the spatial-temporal patterns of neural interactions between recordings sites of multichannel ECoG and LFP data, we utilized ERC (Korzeniewska et al., 2008), a method successfully used over years in studying articulation and word production (Korzeniewska et al., 2008, 2011, 2020, 2022; Nishida et al., 2017; Wang et al., 2021), as well as subthalamic-cortical networks (Alhourani et al., 2020). Using this method, previous studies revealed participation of STN in motor planning, in modulation of ongoing movement, and in somatosensory integration. Therefore, it is a suitable tool to investigate the potential role of STN in speech. Moreover, the advantage of the ERC method is that it does not require any a priori model of network interactions.

ERC is a metric based on Granger causality that is designed to estimate the directionality, intensity, and time course of statistically significant event-related changes in causal interactions or neural activity propagations among recording sites. According to Granger causality, for signal *y* to be considered causally influenced by signal *x*, knowledge of *x*’s past must be able to significantly improve the prediction of *y*’s present (Granger, 1969). To evaluate the causality between more than two time series, as in multichannel electroencephalography (EEG), an MVAR model is fitted to the recorded signals. The model assumes that the value of the *x* at a time *t* depends on its *p* previous values and the random component *e*(*t*). The MVAR process for vector signal *x*(*t*) consisting of multiple signals can be expressed as

$$x\left(t\right)=-\sum_{j=1}^{p}A_{j}x\left(t-j\right)+e\left(t\right)$$ (1)

where the coefficients in each matrix *Aj* are calculated by solving the Yule-Walker equations (Walker, 1931; Yule, 1927). To ensure that the observed interactions are direct ones, not spurious causalities, the ERC method is built upon direct directed transfer function, indicating only direct propagations and excluding the influence of indirect ones as mediated by other recording sites (Blinowska, 2011; Korzeniewska et al., 2003). The intensity and spectral content *z*_*kl*_(*f*, *t*) of the causal influence of channel *l* onto channel *k* (*l* → *k*) is estimated by

$$z_{kl}\left(f,t\right)=\frac{\left|h_{kl}\left(f,t\right)\right|\left|c_{kl}\left(f,t\right)\right|}{\sqrt{\sum_{f}\sum_{kl}{\left|h_{kl}\left(f,t\right)\right|}^{2}{\left|c_{kl}\left(f,t\right)\right|}^{2}}}$$ (2)

where *h*_*kl*_(*f*, *t*) is an element of transfer function matrix, a measure of the *directed* relationship between channel *l* and channel *k*, while *c*_*kl*_(*f*, *t*) is an element of partial coherence matrix, a measure of *direct* relationship between the channels. Therefore *z*_*kl*_(*f*, *t*) shows whether a signal component at a given frequency *f* in channel *k* is shifted in time with respect to a signal component of the same frequency in channel *l*, and whether the shifted components are coherent and are not explained by components of other channels. *z*_*kl*_(*f*, *t*) takes values from 0 to 1. Zero indicates a lack of direct causal relationships. The nonzero values of *z*_*kl*_(*f*, *t*) are interpreted as a flow of activity from channel *l* to channel *k* (*l* → *k*). To follow the temporal course of brief changes in signal propagation between brain regions, while ensuring the local stationarity of the signals, we used an algorithm enabling the estimation of neural activity propagation for multiple realizations of the same stochastic process (i.e., many trials/repetitions of the task) in a short sliding time window (Ding et al., 2000).

To ensure enough data points for MVAR modeling, we estimated the sufficient number of EEG samples by the inequality

$$\frac{K\left(p+1\right)}{N_{s}n_{t}}<0.1$$ (3)

where *K* denotes the number of channels, *N*_*s*_ is the length of the time window (number of samples per one repetition epoch), and *n*_*t*_ is the number of trials/repetitions. To ensure the good fit of MVAR model to recorded signals, the model order was determined using Akaike information criterion (Akaike, 1974).

For each subject and each recording session, signals were segmented into 520 ms of pre-stimulus baseline (longest available epochs of no activity ISI) or 1 s post-stimulus response epochs (long enough to include entire spoken response) aligned to speech onset, implemented in custom analysis interface software (Franaszczuk & Jouny, 2004). MVAR coefficients and the intensities of the causal influence *z*_*kl*_(*f*, *t*) were computed for 140 ms long windows (as indicated by Akaike criterion), shifted by 5.6 ms (to ensure smooth coverage of the analyzed signals).

The short time window algorithm uses all repetitions of an event to provide MVAR estimator for *z*_*kl*_(*f*, *t*), to find a statistical significance of a change in *z*_*kl*_(*f*, *t*) relative to the pre-stimulus baseline. Therefore, we employed a bivariate smoothing model based on two-dimensional moving average to access statistical significance and to ensure precise embedding of the results in the time–frequency space (Korzeniewska et al., 2022). The moving average is optimal for reducing random noise while retaining a sharp step response, it allows precise indication of time of the change in *z*_*kl*_(*f*, *t*), and provides an efficient smoothing estimator for statistical testing (Smith, 1999). Only task-related increases in ERC were analyzed. Decreases in ERC were not taken into consideration because the physiological interpretation of flow decreases during event-related task performance is not straightforward (Korzeniewska et al., 2008, 2011, 2020).

While recordings were collected from four subjects, ERC analyses were conducted over 10 sessions on a session-by-session basis, where STN recordings in different sessions from an individual patient are taken from different regions within the STN. Statistically significant ERC values (as compared with a 520 ms pre-stimulus baseline) for speech-aligned recordings at the level of the single subject were also statistically tested for group significance. A two-sided *t* test was used to test for the null hypothesis of zero differences between ERC means, where the normalizing standard error was the standard deviation of the estimated mean difference. The threshold for statistical significance was set at *α* = 0.05 (95% confidence interval) after using false discovery rate correction to control for multiple comparisons (Benjamini & Hochberg, 1995). A more expansive description of the ERC-specific statistical methods used in this study can be found in Korzeniewska et al. (2022).

The number of analyzed directed connections within and between recording sites in all patients is shown in Table 2.

**Table 2.** Number of analyzed directed connections summed over all patients.

**Pooled words and pseudoword trials**					**Word trials**					**Pseudoword trials**				
Number of directed connections	PreCG	PoCG	STG	STN	Number of directed connections	PreCG	PoCG	STG	STN	Number of directed connections	PreCG	PoCG	STG	STN
PreCG	38	90	0	26	PreCG	18	22	0	16	PreCG	32	76	0	18
PoCG	90	46	48	40	PoCG	22	8	32	22	PoCG	76	44	38	34
STG	0	48	38	26	STG	0	32	24	22	STG	0	38	38	26
STN	26	40	26	0	STN	16	22	22	0	STN	18	34	26	0

*Note*. Connections are all theoretically possible propagations between recording sites revealing task-related activation. PreCG = precentral gyrus (primary motor cortex), PoCG = postcentral gyrus (primary somatosensory cortex), STG = superior temporal gyrus (auditory cortex), STN = subthalamic nucleus.

### Data and Code Availability

All analyses were performed, using either Psychophysics Toolbox extensions (Brainard, 1997) or in-house developed code, and implemented in MATLAB (MathWorks, 2018). All code and the data for the behavioral and the ERC analyses are available upon request.

## RESULTS

### Behavioral Responses

A lexicality effect was observed for several aspects of the speech response (Table 3). The mean reading latency for word trials was statistically shorter than the latency during pseudoword trials (*p* < 0.001, Wilcoxon signed-rank tests), suggesting that pseudowords require additional or encumbered processing and thus require longer to read (Frankish & Turner, 2007; Grainger et al., 2003; Khateb et al., 2014). The duration of articulation of CVC stimuli phonemes were also calculated from audio recordings. The mean duration of both the first (C1) and second (C2) consonants of word trials were significantly shorter (*p* = 0.001 and *p* = 0.008, respectively) than those of pseudoword trials. The mean duration of vowel articulation was significantly longer (*p* = 0.007) for word trials than for pseudoword trials. The mean duration of the subjects’ spoken responses was significantly shorter for words (*p* = 0.02). Calculated speech latencies were used in subsequent analyses to ground the event triggers for speech onset, and the average duration of C1, V, and C2 articulation was used as reference epochs (as shown in Figure 3, below, and Figure 6 in Reading Words vs. Pseudowords).

**Table 3.** Latencies of speech onset in the reading tasks and durations of first consonant (C1), vowel (V), and second consonant (C2), shown as means ± *SEM*.

	Speech latency (ms)	First consonant (C1) duration (ms)	Vowel (V) duration (ms)	Second consonant (C2) duration (ms)
Words	736 ± 34***	93 ± 3**	217 ± 5	170 ± 5*
Pseudowords	818 ± 50	105 ± 4	203 ± 4**	187 ± 5
Pooled words and pseudowords	795 ± 31	97 ± 3	207 ± 4	177 ± 5

*Note*. Statistical differences between words and pseudowords were calculated using Wilcoxon signed-rank tests, with the significantly shorter of a pair denoted by number of asterisks.

* *p* < 0.05. ** *p* < 0.01. *** *p* < 0.005.

### Subthalamic-Cortical Interactions During Reading Aloud

**Figure 2.**
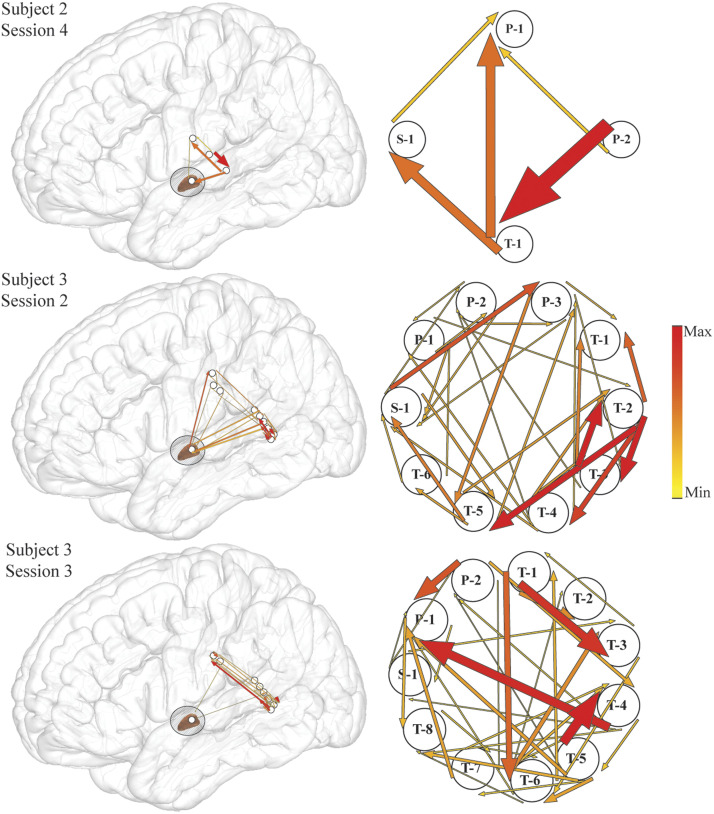
Examples of event-related causality (ERC) of high-gamma propagation integrated over the −500 to −300 ms time interval prior to speech onset (the time interval of significant increase in high-gamma propagation from superior temporal gyrus to subthalamic nucleus for these subjects) for pooled pseudoword and word trials plotted over a lateral view of the brain (left panel) and their enlarged schematic representation (right panel). Arrow width and color both correspond to the strength of ERC propagation. Only sites used for estimating ERC flows are shown, and 15% of the smallest ERCs are not shown for clarity. Labeled electrodes: S = subthalamic nucleus, P = primary somatosensory cortex, T = superior temporal gyrus.

**Figure 3.**
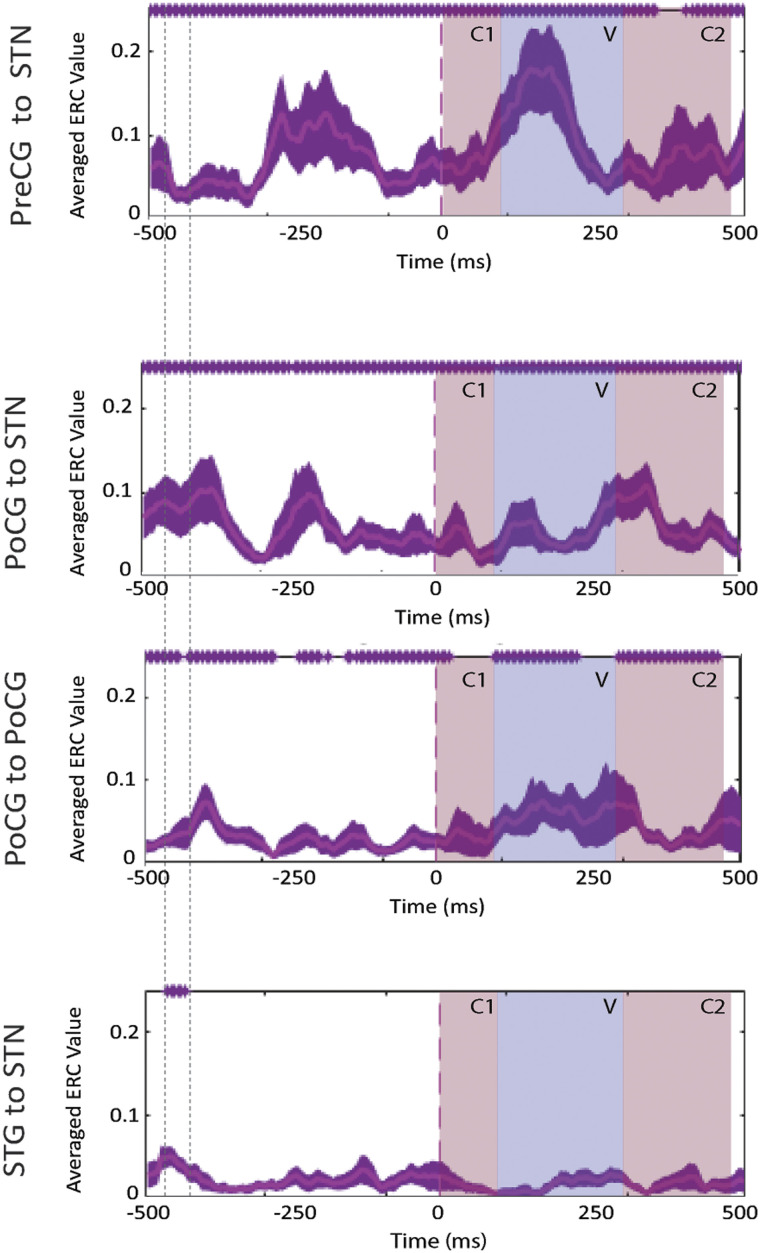
Event-related causality (ERC) as a function of time for high-gamma activity propagation between the subthalamic nucleus (STN) and cortical recording sites, averaged over pooled word and pseudoword trials of all patients. Purple traces represent mean ERC with confidence intervals (aligned to speech onset *t* = 0 ms). Purple asterisks (*) along the top of each plot indicate time points of significant increases in high-gamma propagation as compared to pre-stimulus baseline. Colored time intervals denote the average durations of consonants (C1 and C2) and vowel (V) articulation. Interactions that exhibited no significant increases in ERC at the level of group-pooled statistics at any point of the time period are not included. (ERC propagation integrated over the time period denoted by vertical dotted lines is schematically represented in Figure 7 in the Discussion.) PreCG = precentral gyrus, PoCG = postcentral gyrus, STG = superior temporal gyrus.

We used ERC to estimate the directionalities and magnitudes of statistically significant increases in propagation of high-gamma activity between STN and cortical recording sites, with respect to speech onset (time = 0 ms). Because an MVAR model encompassing all recorded signals would include signals unrelated to the investigated process, analyses were limited to sites revealing event-related augmentation of spectral power. We observed statistically significant increases in high-gamma propagation between STN and all studied cortical areas over the course of the overt reading task in pooled (combined word and pseudoword) trials, at the level of single sites in individual subjects’ sessions (Figure 2), as well as in group analyses (Figure 3), suggesting sustained cortical-STN engagement over the majority of the task. Figure 2 shows that the significance of ERC flows does not result merely from the grouping of results across subjects. All regions of interest displayed significant, reciprocal interactions with the STN at the level of the individual subject. These flows are asymmetric on a session-by-session basis, depending on the recording location within the STN. Flows are greatest between recording sites within STG, and we observed numerous and varied parallel interactions between the STG and primary somatosensory cortex, both of which were anticipated from our speech task (Hamilton et al., 2021). Notably, interactions between the STG and STN are significant at the individual subject level at approximately 400 ms prior to speech onset (three examples shown in Figure 2), and were in one subject reciprocal (Figure 2, middle row).

The results of the group-level pooled (words and pseudoword trials) ERC analysis is shown in Figure 3. Aligning to speech onset revealed significant (*p* < 0.05) unidirectional propagations from precentral gyrus to STN, peaking 250 ms before speech onset (Figure 3, top), followed by a second, larger increase in propagation midway (approximately 250 ms) through articulation of the vowel phoneme. Significant reciprocal interactions between STN and postcentral gyrus were likewise observed throughout almost the whole duration of the trial, with significant flows originating in STN aligning approximately with the onset and subsequent duration of V and C2 articulation (Figure 3, center). As observed at the individual subject level, at the group level, a short duration propagation (∼50 ms) from STG to STN preceded speech onset by approximately 450 ms (Figure 3, bottom; see also Figure 7 in Discussion).

### Reading Words vs. Pseudowords

**Figure 4.**
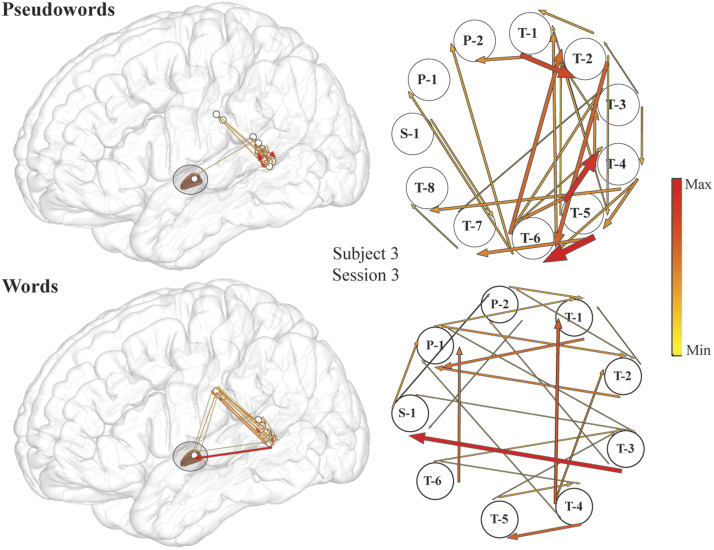
An example of stronger high-gamma propagation from superior temporal gyrus (STG) into subthalamic nucleus (STN) during word compared to pseudoword reading in individual subject (Subject 3, Session 3). Event-related causality (ERC) of high-gamma propagation integrated over −160 to −15 ms time interval (the time interval of significant increase in high-gamma propagation from STG to STN for this subject) for pseudowords (top) and words (bottom) plotted over a lateral view of the brain (left panel) and their enlarged schematic representation (right panel). Arrow width and color both correspond to the strength of ERC propagation. Only sites used for estimating ERC flows are shown, and 15% of the smallest ERCs are not shown for clarity. Labeled electrodes: S = STN, P = primary somatosensory cortex, T = superior temporal gyrus.

**Figure 5.**
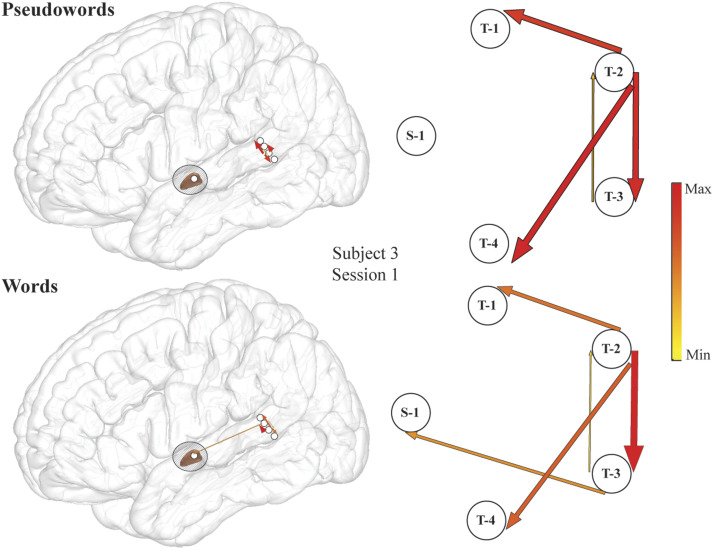
An example of high-gamma stronger propagation from superior temporal gyrus (STG) into subthalamic nucleus (STN) during word compared to pseudoword reading in individual subject (Subject 3, Session 1). Event-related causality (ERC) of high-gamma propagation integrated over −350 to −200 ms time interval (the time interval of significant increase in high-gamma propagation from STG to STN for this subject) for pseudowords (top) and words (bottom) plotted over a lateral view of the brain (left panel) and their enlarged schematic representation (right panel). Arrow width and color both correspond to the strength of ERC propagation. Only sites used for estimating ERC flows are shown, and 15% of the smallest ERCs are not shown for clarity. Labeled electrodes: S = STN, P = primary somatosensory cortex, T = superior temporal gyrus.

**Figure 6.**
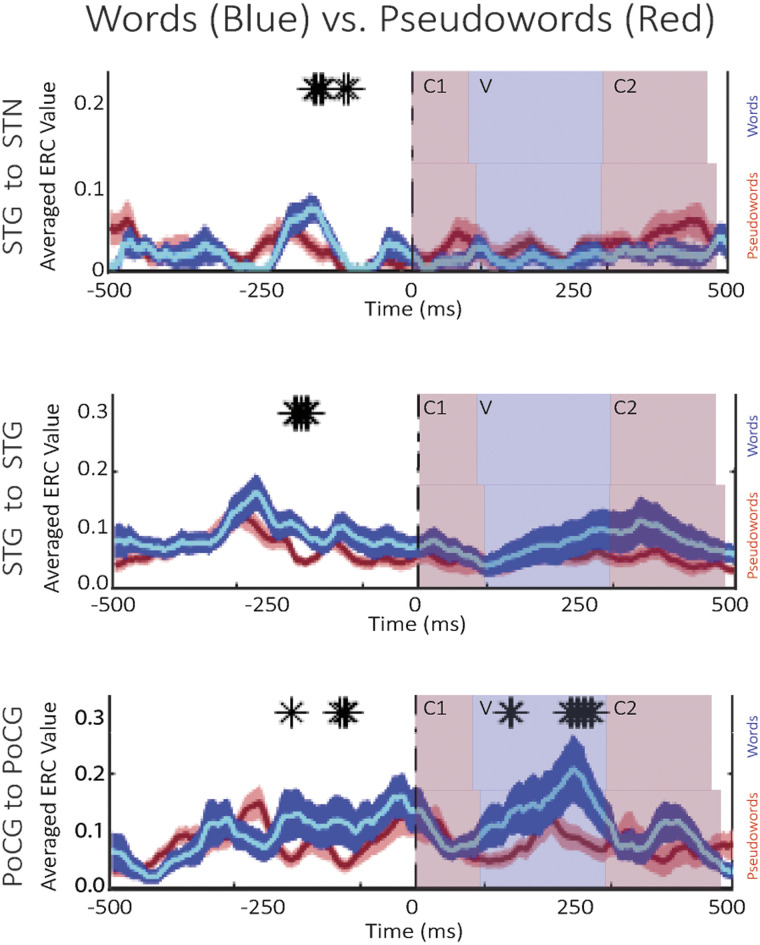
Event-related causality (ERC) as a function of time for high-gamma activity propagation, averaged over word (blue traces) and pseudoword trials (red traces) of all patients. Black asterisks (*) denote significant differences between word trials and pseudoword trials. Colored time intervals denote the average durations of consonants (C1 and C2) and vowel (V) articulation. (ERC propagation integrated over the time period of pre-speech significant differences is schematically represented in Figure 8, below.)

Further exploration of STG–STN interactions revealed significant lexicality effects in high-gamma propagations on both the single patient level (Figure 4 and Figure 5) and the group-analysis level (Figure 6) from STG to STN prior to the start of articulation, with greater propagation when subjects read words than when they read pseudowords. From recordings on the individual patient level, we observed mixed effects of lexicality on these interactions (Figure 4 and Figure 5). Depending on where the recording sites were located within STN, reciprocal, albeit uneven, propagations were observed between the STG and STN during word reading, noting that ERC analysis displays only significant (*p* < 0.05) flows (Figure 4, bottom row). A second region in the STN exhibited flows from STG to STN during word reading (Figure 5, bottom row), and exhibited no significant flows at all during pseudoword reading (Figure 5, top row). Note that individual subject figures (Figure 2, Figure 4, and Figure 5) may display significant flows at time epochs that do not show significance at the group level (Figure 3 and Figure 6). By definition, ERC is tested using the statistical method and approach as shown in Korzeniewska et al. (2022). For a single subject we used all selected trials to obtain one short direct directed transfer function (SdDTF) value for words, and one SdDTF value for pseudowords per time–frequency point, thus it was only possible to compare these values to the baselines, containing more time points in one frequency. We tested significance of ERC at the group level (Figure 6), which revealed significantly greater propagations from the STG to the STN during word reading, approximately 150 ms prior to speech onset. Immediately preceding this observation (approximately 50 ms earlier relative to speech onset), significantly greater neural interactions during word versus pseudoword trials *within* the STG were observed at the group level (Figure 6, center) and at the individual patient level (Figure 5), supporting the STG’s role in lexical processing (Simos et al., 2000; Simos et al., 2002). These data suggest that information transfer from auditory cortex to the basal ganglia is important during reading aloud and is related to the lexicality of what is read. The lexicality-modulated propagations occurred at a much later time point than the STG to STN interactions observed in the pooled analyses, perhaps suggesting that we are observing two different phenomena. The earlier neural flow, visible for the pooled data analysis, did not pass the significance level when trials were differentiated by lexicality (and trial numbers were reduced accordingly). Propagations within primary motor cortex were also significantly greater for word trials than for pseudoword trials before and after speech onset (Figure 6, bottom), particularly during vowel articulation, which was not observed for propagation between precentral gyrus and STN.

## DISCUSSION

**Figure 7.**
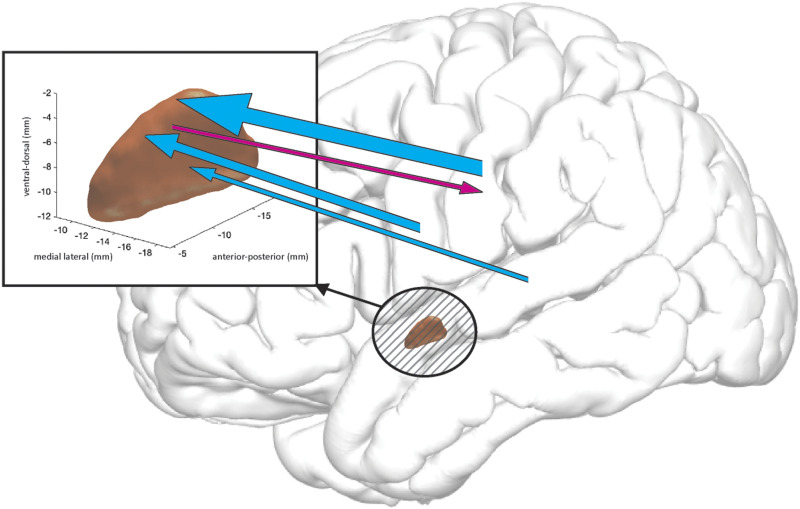
A schematic of high-gamma propagation integrated over −450 to −400 ms prior to speech onset (denoted by vertical dotted lines in Figure 2)—that is, the interval of significantly stronger propagation from superior temporal gyrus to subthalamic nucleus (STN), as compared to pre-stimulus baseline. The width of the arrows represents the magnitudes of increases in high-gamma propagation, as compared to pre-stimulus baseline. Magenta = from STN into cortex, cyan = from cortex into STN. Only statistically group-significant increases in high-gamma propagation are shown.

Speech involves cognitive and motor processes across a large system of cerebral areas. The basal ganglia has been implicated in speech processing, although the manner in which subcortical sites, including the STN, participate in speech networks is still poorly understood. Our findings provide evidence of sustained, and at times reciprocal, propagation (ERC flows) of high-gamma (60–180 Hz) activity between cortical language areas and the STN during a spoken word production task at the single recording session, subject, and group levels, which is schematically represented in Figure 7. Moreover, our findings suggest that STG, primary motor cortex, and primary somatosensory cortex activity directly influence STN, and that the lexicality of read words and pseudowords modulates this influence prior to the onset of articulation. These findings were substantiated through ERC’s emphasis on direct network node interactions.

Psycholinguistic models have identified specific cognitive lead-in processes that may precede the motor processes driving articulation (Coltheart et al., 1993, 2001; Coltheart & Rastle, 1994; Guenther & Vladusich, 2012; Gupta & Macwhinney, 1995; Indefrey, 2011; Indefrey & Levelt, 2000, 2004; Price et al., 1997; Quaglino et al., 2008; Schomers & Pulvermüller, 2016). Importantly, many of these cognitive processes are thought to be influenced by whether the to-be-produced utterance is a known word (and therefore also a familiar motor sequence) or a pseudoword (a pronounceable but novel motor sequence). For instance, to generate a phonological representation for a visually presented word, the dual route perspective of reading proposes that while real words can be read aloud through access to the mental lexicon, the pronunciation of written pseudowords must be created by grapheme-to-phoneme conversion (Coltheart et al., 1993, 2001; Woolnough et al., 2022). The resulting novel phonological representations likely require additional processing in posterior STG before their phonetic articulatory realization, or at least incur an encumbered lexical search therein (Indefrey, 2011; Indefrey & Levelt, 2004). It therefore takes longer to plan the production of pseudowords, as reflected in elevated response latencies (Table 3) (Frankish & Turner, 2007; Grainger et al., 2003; Khateb et al., 2014). Interestingly, even after a phonetic articulatory realization has been achieved, lexicality effects can still be at play. For instance, errors in covert speech show a lexicality bias, which has been attributed to the greater difficulty in internally monitoring and repairing impending speech errors that are lexically valid (e.g., Spoonerisms such as saying “darn-bore” instead of “barn-door”).

The complexity of cognitive processes that occur prior to articulation makes a definitive interpretation of our results premature. However, they are consistent with the speculations of Jorge et al. (2022), who hypothesized that the hyperdirect STG–STN pathway could play an important role in the feedforward control of speech. This hypothesis was based on predictions from speech production models and general theories about basal ganglia function. For instance, the gradient order directions into velocities of articulators (GODIVA) computational model (Guenther & Hickok, 2015a, 2015b) hypothesizes that projections originating in the supplementary motor areas and passing through the basal ganglia serve as gates on the outflow of motor commands (Albin et al., 1989; Guenther & Hickok, 2015a; Pickett et al., 1998). These projections provide GO signals that signal speech motor patterns to be put into action by primary motor cortex and implicate the basal ganglia in the planning and motor loops involved with both articulation and speech motor program learning. The GODIVA model further hypothesizes that the basal ganglia is involved in cortical initiation of phonological representations across the entire articulatory arc, from phoneme-level motor programs within larger syllabic and supra-syllabic motor sequences that drive articulatory movements during speech, to the chunking of isolated movements into the action sequences that permit the achievement of particular articulatory goals (Bostan et al., 2010, 2013; Guenther & Hickok, 2015a; Peeva et al., 2010). Our results suggesting a feedforward gamma propagation to the STN that peaks before shifts in articulation, including the onset and offset of speech and phoneme transitions (Figure 3), generally supports these concepts.

The interactions we observed between STG and STN are also consistent with another aspect of the GODIVA model, which uses the auditory context from perception of one’s own utterance to determine the precise instant in time to initiate a motor program as the utterance is concluding (Guenther et al., 2006; Guenther & Hickok, 2015a, 2015b). Thus, signals from the auditory cortex to the basal ganglia might include both auditory state and auditory error signals. An alternative explanation may be that the STG is responsible for storing phonological representations, projecting auditory input to premotor cortex to help develop speech sound maps. Through this route, the STG and its speech sound map are predicted to be engaged during both production (in a self-monitoring capacity) and perception of acquired speech sounds, consistent with the dual stream model (Eckers et al., 2013). Periods of elevated propagations from STG to STN may reflect the STG’s role in phonological form access or word retrieval, which are also necessary for self-monitoring (Gow, 2012; Heim et al., 2013). These results are consistent with the hypothesis that the basal ganglia are involved in self-monitoring (error prediction, evaluation, and corresponding behavioral compensation mechanisms) in motor and cognitive contexts (Dabney et al., 2020; Glimcher, 2011; Mikhael & Bogacz, 2016; Morita & Kawaguchi, 2015; Schultz, 2016).

**Figure 8.**
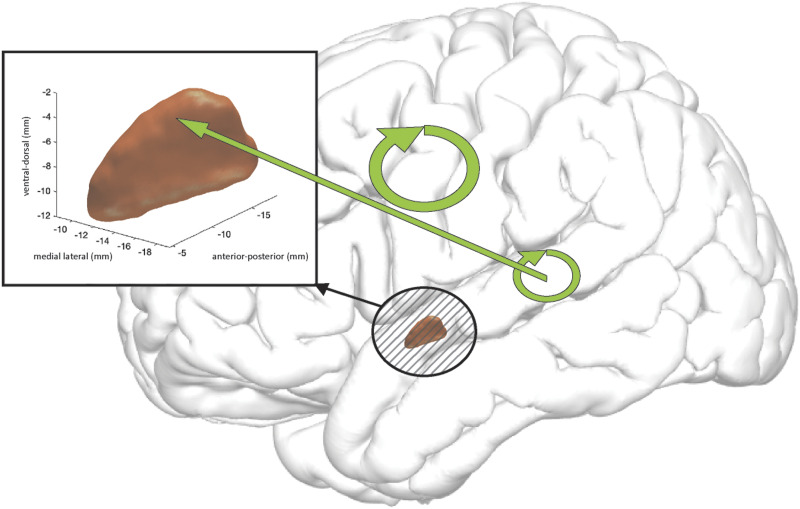
A schematic of a difference in intensity of high-gamma propagation for words vs. pseudowords, integrated over −208 to −117 ms prior to speech onset, as denoted by earliest and latest time points of statistical significance (black asterisks in Figure 6). The width of the straight and circular arrows represents increases in magnitudes of high-gamma propagation for words as compared to pseudowords. The straight arrow denotes propagation from superior temporal gyrus (STG) into subthalamic nucleus. Circular arrows denote propagations between recording sites located within STG and within primary motor cortex accordingly.

We also found that STN activity was directly influenced by propagations from the STG approximately 150 ms before the start of articulation (Figure 4, bottom row, and Figure 6, top). This timing aligns with the expected activation of the initiation map of GODIVA’s feedforward control subsystem (Guenther & Hickok, 2015a). Additional evidence for the basal ganglia’s influence in this late stage of articulatory preparation comes from patients with PD, who have difficulty executing voluntary movements and often exhibit hypokinetic dysarthria, characterized by speech freezing and reduced speech volume (Duffy, 2012). Importantly, STG propagations to the STN at this later time point were greater for words than for pseudowords (Figure 6, top, and Figure 8). This could reflect the STG’s encoding of lexical structure. Perhaps, as subjects read each word, they activate a phonological output lexicon within the STG and load an existing, established acoustic representation, which, in turn, influences the STN’s gating of the associated motor mapping (Gvion & Friedmann, 2016; Simos et al., 2000; Simos et al., 2002). Increasing the output of the STN subsequently increases the output of the basal ganglia, and ultimately acts to inhibit the motor cortex, so this result may be interpreted as word-reading-related motor gating initiated by the STG, which is not cued when pseudowords are encountered. Since we previously showed that gamma activity in the STN was greater for the pseudoword condition, other cortical regions, such as the inferior frontal gyrus, may have greater influence on the STN during pseudoword production. Indeed, Nozari’s (2020) view on production monitoring are consistent with the idea that lexical-level representations may contribute to speech monitoring. Further, this notion of gating by the STN during speech production, where one might expect greater conflict for nonwords as compared to words, would result in higher thresholds (and thus speech latencies such as in the results shown in Table 3) for the initiation of speech production. Essentially, Nozari stated that conflict monitoring (Botvinick et al., 2001) is based upon the idea that in situations with one clearly correct response one representation will have a higher activation than others, and conflict occurs when several representations have similar levels of activation, resulting in higher likelihoods of generating errors (Nozari, 2020). The natural dynamics of the speech production system, such as the mapping of semantic representations to lexical items by the STG, result in conflict (Nozari et al., 2011), and decisions regarding how much conflict is high enough to be detected as an error is a task nicely suited to a decision making framework (Nozari, 2018). Nozari proposed that the information generated during primary production processes can be used to gauge the probability of speech production errors, and that the probability of an error could be reduced by applying appropriate control. The STN fits nicely into such a role, potentially by acting to delay production until the mapping processes have converged more closely on a certain representation, allowing moment-by-moment adjustments to speech plans to optimize performance (Nozari, 2020). When competition at the lexical-level is high, conflict signals by the STN, in conjunction with the STG, may prolong the selection process until conflict falls below a certain level through competitive selection (Roelofs, 2004), similar to what we see in our speech latencies in Table 3. This conflict-based model also cleanly complements the goal of adjustment of speech performance defined by Levelt’s theories of speech monitoring (Levelt, 1983).

Additionally, the observed lexicality effects may suggest the involvement of the STN, and its interaction with cortical sites, in the acquisition and processing of new motor plans for articulatory gestures, as our pseudowords require the unpracticed production of a syllable (and thus decreased output from the basal ganglia to permit it) and linguistic models consider speech processing to be chunked at the syllable level (Segawa et al., 2019). The direct and indirect pathways of the basal ganglia have been proposed to mediate a competition that selects the proper movement among competing alternatives (Mink, 1996). We posit that the STN could play a dual modulatory role in this framework. First, the STN may generate a braking signal for rapid inhibition of unwanted (particularly in the case of known words) chunked movements by providing diffuse excitatory input to the basal ganglia output nuclei, thereby inhibiting motor programs in the cortex via inhibition of the thalamus. Second, the STN may interact with the globus pallidus to modulate motor programs involved in a given speech sequence, such as scaling selected movements and signaling the imminent completion of a given movement. We showed that STN propagations to primary motor cortex peaked prior to the start of articulation, consistent with the idea that the STN may participate in selecting articulatory gestures or stored syllabic plans. Interestingly, STN to primary motor cortex propagation was not significantly different for words versus pseudowords. While one might have expected to see articulation-related differences in propagation from the STN, overly conservative statistical corrections for multiple comparisons or the study’s low trial and electrode count may have been too limiting. Novel strings of articulatory gestures (such as those produced in response to nonword or pseudoword stimuli) have previously been shown not to elicit enhanced neural activity in primary motor cortex, so that may be also reflected here in primary motor cortex interactions with STN (Flinker et al., 2015).

Our ERC results further substantiate a role for the STN generally in sensory processing, suggesting that sequential neural interactions in gamma activity between primary somatosensory cortex and STN occur just before speech onset (Figure 3, center). Recent studies support the idea that the STN is a hub for the integration of sensory information within the cortical-basal ganglia network (Accolla et al., 2014, 2016; Haynes & Haber, 2013; Lambert et al., 2012). The bidirectional neural interactions between the STN and primary somatosensory cortex in the gamma frequency range are consistent with evidence from STN-to-cortical spike-phase coupling and ERC analyses during hand-gripping, that suggest the presence of information transfer between sensory cortex and the STN (Alhourani et al., 2020; Lipski et al., 2018).

There are aspects of our data that limit interpretation of the ERC results. It is important to note that the implantation of STN electrodes in our subjects was performed solely according to clinical needs. Therefore, the number of STN sites revealing ERC were limited by the electrode locations unique to each session in each subject. This limitation was mitigated by using group statistical analyses subsequent to statistical analyses in single patients (Korzeniewska et al., 2022). However, it is possible that the STN is strongly influenced by activity in other cortical areas during production of pseudowords, such as the inferior frontal gyrus, where a recent study revealed increased activation to pseudowords compared to words (Flinker et al., 2015). We were unable to investigate the mediating effects of other cortical sites (e.g., inferior frontal gyrus, supplementary motor area, or ventral precentral gyrus) on STN activity in this study because research electrodes were not present in more anterior cortical regions. Additionally, our results were collected from patients with PD, a disease that affects auditory processing of voice and speech, and thus the extent to which these findings represent interactions in the non-PD brain is unknown. PD has been found to alter the statistical relationship between LFP phase and spike timing in global brain networks and, as many brain functions are known to depend on this mechanism for task performance, its disruption in the cortical-basal ganglia motor circuit could result in pathological impairment of articulation in our subjects (Fries, 2005; Gatev & Wichmann, 2009; Goldberg et al., 2004; Li et al., 2012; Shimamoto et al., 2013). Previous studies of STN LFPs have suggested the possibility of high frequency phase-amplitude interactions as a PD biomarker, which, given our interest in the high-gamma frequency band, may be impacting our results (López-Azcárate et al., 2010; Özkurt et al., 2011). PD patients also exhibit excessive cortical coupling of the phase of beta activity to gamma amplitude (de Hemptinne et al., 2013, 2015). However, correlation of beta and gamma power with PD symptom severity was not significant in these patients (Chrabaszcz et al., 2019, 2021; Dastolfo-Hromack et al., 2022). Moreover, beta activity decreases dramatically during GO responses (Zhang et al., 2008), resulting in an absence of detectable beta activity propagation and making physiological interpretation of beta ERC difficult (Korzeniewska et al., 2008, 2011, 2020). Taking these constraints into account, we did not perform the ERC analysis on beta band activity.

In summary, this unique study of simultaneously recorded cortical and subthalamic activity during reading aloud demonstrates that high-gamma activity propagates between auditory cortex and the STN, supporting the idea that the STN is a hub, dynamically integrating sensory information for speech and other motor plans. Understanding the role of the cortical-basal ganglia network in speech production will improve models of speech motor control and may lead to more effective treatments for neurological speech disorders.

## ACKNOWLEDGMENTS

We thank Dr. Witold Lipski for his help with data acquisition, and Noah Lu for his help with data preprocessing.

## FUNDING INFORMATION

Robert M. Richardson, National Institute of Neurological Disorders and Stroke (https://dx.doi.org/10.13039/100000065), Award ID: U01-NS098969. Nathan E. Crone, National Institute of Neurological Disorders and Stroke (https://dx.doi.org/10.13039/100000065), Award ID: R01-NS091139. Robert M. Richardson, National Institute of Neurological Disorders and Stroke (https://dx.doi.org/10.13039/100000065), Award ID: U01-NS117836.

## AUTHOR CONTRIBUTIONS

**Alexander R. Weiss**: Formal analysis; Methodology; Software; Visualization; Writing—original draft; Writing—review & editing. **Anna Korzeniewska**: Conceptualization; Formal analysis; Methodology; Software; Writing—original draft; Writing—review & editing. **Anna Chrabaszcz**: Data curation; Software. **Alan Bush**: Data curation; Investigation. **Julie A. Fiez**: Conceptualization; Writing—review & editing. **Nathan E. Crone**: Conceptualization; Funding acquisition; Writing—review & editing. **Robert M. Richardson**: Conceptualization; Funding acquisition; Writing—review & editing.

## TECHNICAL TERMS

Basal ganglia: A group of subcortical nuclei associated with a variety of functions including control of motor movements, cognition, and emotion.
Parkinson’s disease (PD): A degenerative disorder that mainly affects the motor system characterized by the death of dopamine-producing cells in the substantia nigra.
Deep brain stimulation (DBS): A neurosurgical procedure involving the placement of electrodes into the brain that treats disease symptoms through electrical stimulation.
Subthalamic nucleus (STN): Part of the basal ganglia associated with action selection. Deep brain stimulation of the STN is used to treat patients with Parkinson’s disease.
Superior temporal gyrus (STG): Part of the brain’s temporal lobe responsible for the sensation of sound and processing of speech.
Event-related causality (ERC): A statistical method that estimates the strengths and directionalities of neural activity propagations between nodes in a network.
Multivariate autoregressive model (MVAR): A modeling technique that recognizes patterns in time series data. The output variable depends on its previous values.
Granger causality: A statistical hypothesis test for determining whether one time series’ past results are able to forecast another series’ future results.
Electroencephalography (EEG): A method that uses electrodes to record voltage fluctuations within and around neurons in the brain.
